# Synthesis and Intramolecular Charge Transfer Studies on *meso*-Tetracyanobutadine-Functionalized Diphenylporphyrin Complexes Incorporating Copper and Nickel Metals

**DOI:** 10.3390/molecules31060934

**Published:** 2026-03-11

**Authors:** Sumit Kumar Yadav, Jatan K. Sharma, Muniappan Sankar, Francis D’Souza

**Affiliations:** 1 Department of Chemistry, Indian Institute of Technology Roorkee, Roorkee 247667, India; yadavsumit404@gmail.com; 2 Department of Chemistry, University of North Texas, 1155 Union Circle, #305070, Denton, TX 76203-5017, USA; jatansharma@my.unt.edu

**Keywords:** push-pull porphyrins, excited ultrafast charge transfer, femtosecond transient spectroscopy, cyclic voltammetry, DFT calculations

## Abstract

This study presents the synthesis and electrochemical characterization of *meso*-tetracyanobutadiene (TCBD)-functionalized diphenylporphyrin (DPP) complexes incorporating copper (Cu) and nickel (Ni) metals. These push–pull metallo diphenylporphyrin–TCBD complexes were synthesized via a [2 + 2] cycloaddition–retroelectrocyclization reaction between 5-bromo-15-formyl-10,20-diphenylporphyrin metal(II) complexes (M = Cu, Ni) and tributyl(phenylethynyl)stannate, followed by tetracyanoethylene (TCNE) addition. The resulting TCBD-functionalized porphyrins were obtained in moderate yields (70–75%) and thoroughly characterized by ^1^H and ^13^C NMR, UV-Vis spectroscopy, MALDI-TOF-MS, and single-crystal XRD. Although the single-crystal X-ray structure of NiDPP was solved, DFT calculations were used to determine the structures of the donor–acceptor MDPP-TCBD systems and to visualize their electronic structures. HOMO on the porphyrin π system and LUMO on the TCBD entity were observed, and energy level diagrams clearly laid out the electron donor and acceptor parts of the molecular systems. As expected, these novel donor–acceptor porphyrinoid assemblies exhibited enhanced push–pull properties in both the ground and excited states. Femtosecond transient absorption studies revealed that both **NiDPP-TCBD** and **CuDPP-TCBD** populate the charge-transfer state upon photoexcitation, with lifetimes of 383.1 ps and 484.7 ps, respectively, in benzonitrile. The charge-transfer states populated the triplet or doublet states (in the case of CuDPP) before returning to the ground state.

## 1. Introduction

In recent years, there has been growing scientific interest in the design and characterization of electron donor–acceptor (D-A) systems that exhibit photoinduced charge separation. This process is critical to understanding natural photosynthesis, which converts solar energy [1]. The most beneficial electron acceptor dienophiles are tetracyanobutadiene (TCBD) and tetracyanonaphthoquinone (TCNQ), which easily form strong donor–acceptor (D-A) complexes with the molecules that have been functionalized with electron-rich alkynes through [2 + 2] cycloaddition–retroelectrocyclization reaction, developed by Diederich [2,3]. They are well recognized for exhibiting properties such as strong intramolecular charge transfer (ICT), significant thermal stability, nonlinear optics, and a rich array of electrochemical redox behaviours [4,5,6,7,8]. The magnetic characteristics of some TCBD/TCNQ adducts are intriguing and exhibit high electrical conductivity [9,10,11]. Given these attributes, the exploration of TCBD and TCNQ in advanced electrical and photonic materials research is compelling [2,12].

In materials chemistry, there has been considerable interest in π-extended porphyrins, corroles, and other optical limiters owing to their intriguing nonlinear optical properties. These materials are promising candidates for energy-harvesting applications [13,14,15,16,17]. There are a few studies on donor–acceptor (D-A) porphyrinoid assemblies with TCBD and TCNQ [18,19,20,21,22,23,24,25,26,27,28,29,30,31,32,33,34,35,36,37,38], in which the porphyrin moieties act as dienes due to their electron-rich nature, and the TCBD unit acts as a dienophile due to its electron-deficient nature, resulting in advantageous NLO properties [18,39,40,41]. Gryko and colleagues were the first to report a cycloaddition process that yields mono/bis and extended-TCBD derivatives on *meso*-diphenylethynyl-modified porphyrins, achieving high yields in the synthesis [19]. Gao and coworkers investigated the NLO properties and self-assembly behavior of *meso*-TCNE-appended porphyrins [20,21]. The Misra group reported the fabrication of push–pull porphyrins with functionalization that exhibit charge-transfer interactions and investigated the role of porphyrins in the charge-separation process [22]. Recently, our group demonstrated the optical limiting threshold for *β*-TCBD-functionalized porphyrin moieties, and they emphasized that these porphyrins serve as a better chromophore for optical sensors and shields from exotic alloys [18], and π-extended corroles demonstrated charge stabilization [23], highlighting their crucial role in stabilizing the charge transfer state in Cu and Ag corrole push–pull systems with the singlet excited state. Nickel and copper have already proven their potential in electrocatalysis with diphenyl *meso* substituents. Xianyang et al. recently reported a copper porphyrin with *meso*-(o-carbene) substituents that significantly enhances the electrocatalytic properties for the hydrogen evolution reaction [31]. In this regard, fused Nickel porphyrins are known for their electrocatalytic properties for the oxygen evolution reaction [32,33].

**Chart 1:**
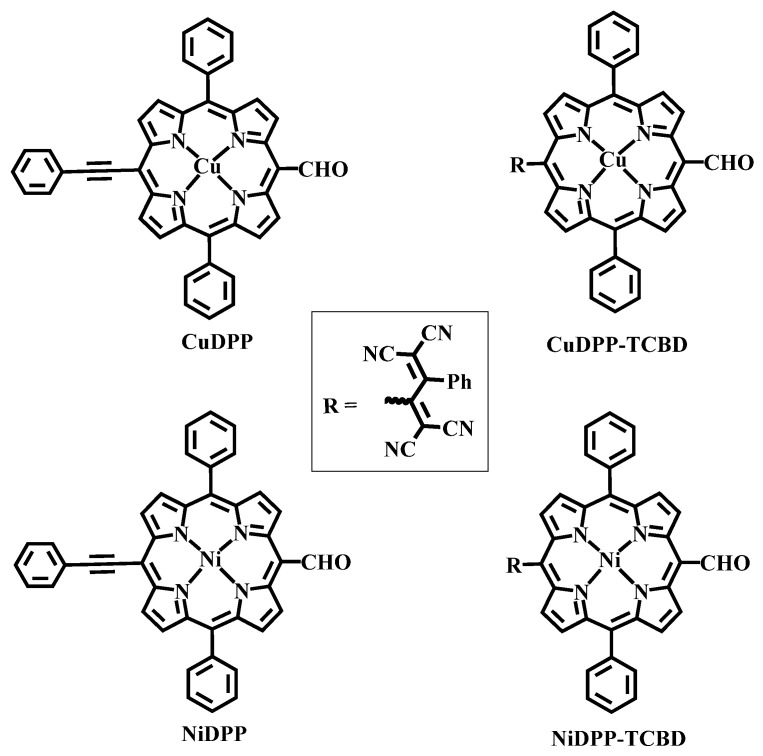
Structures of synthesized precursors and the products.

Over the past two decades, *meso*-arylethynylporphyrins have attracted researchers in materials chemistry owing to their unique optical properties [16]. Moreover, these structures have shown promise in a variety of applications, including photonic limiters, biological imaging, and dye-sensitized solar cells (DSSCs) [24,25,26]. As part of our efforts to build and study this class of push–pull systems, we report herein the synthesis and physicochemical properties of MDPP-TCBD (M = Cu, Ni), wherein the TCBD moiety is at the *meso* position (Chart 1). In the present study, TCBD units were incorporated by adding TCNE to the A2B2 porphyrins, following the methodology reported by Gryko and co-workers [19]. Both NiDPP and CuDPP are weak or non-fluorescent molecules, and a strong electron acceptor, TCBD, is attached at the *meso* positions to create new push–pull systems. The ability of these push–pull systems to undergo charge transfer in both the ground and excited states is the main focus of this study.

## 2. Results and Discussion

### 2.1. Synthesis and Characterization

**Scheme 1:**
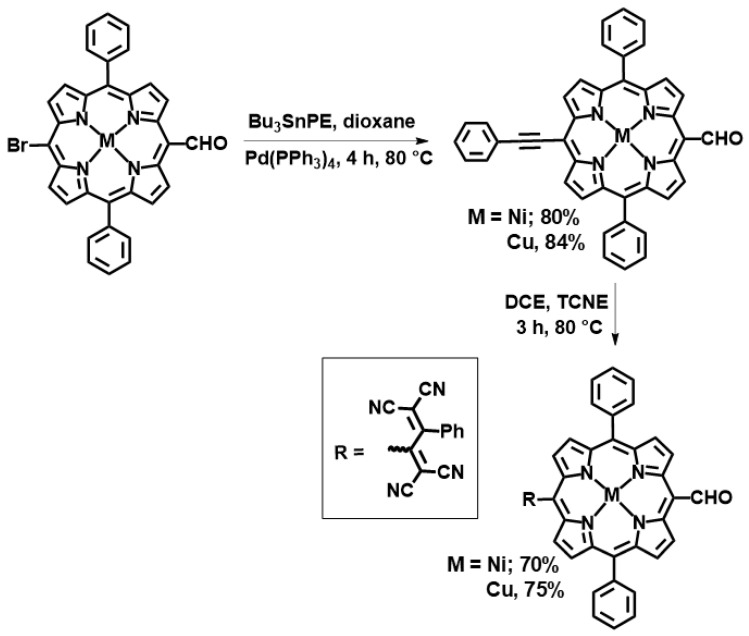
Synthesis of diphenylporphyrin–TCBD conjugates.

**Figure 1:**
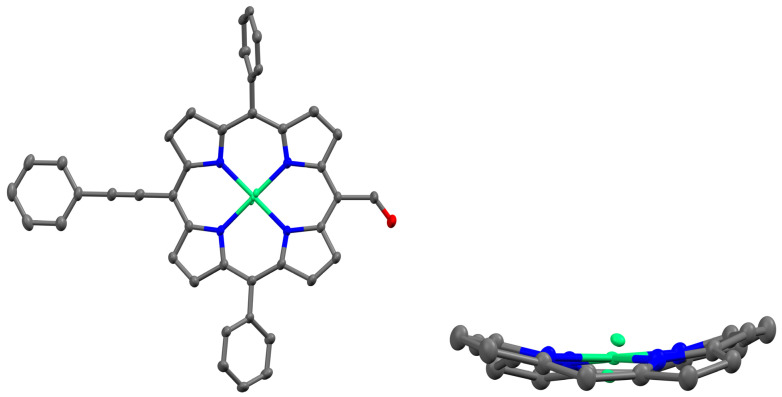
X-ray structure showing the top and side views of **NiDPP** (in the side view, the *meso*-phenyl groups and hydrogen atoms are removed for clarity).

**Figure 2:**
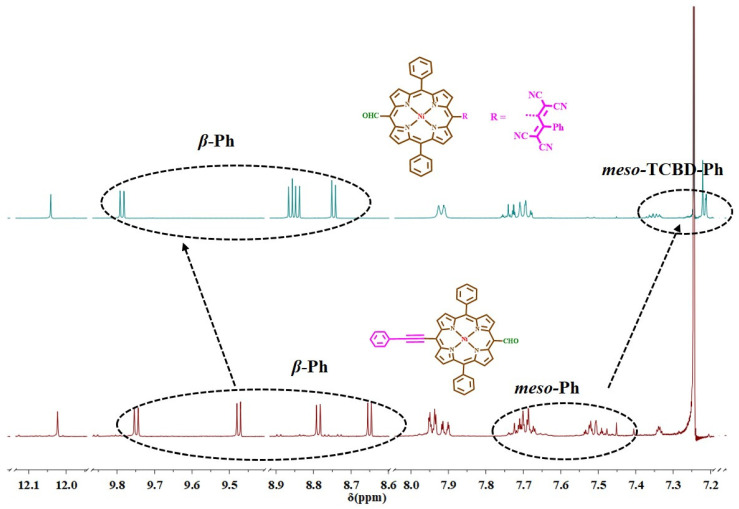
Comparative ^1^H NMR spectra of **NiDPP-TCBD** and **NiDPP** in CDCl_3_ at 298 K.

**Figure 3:**
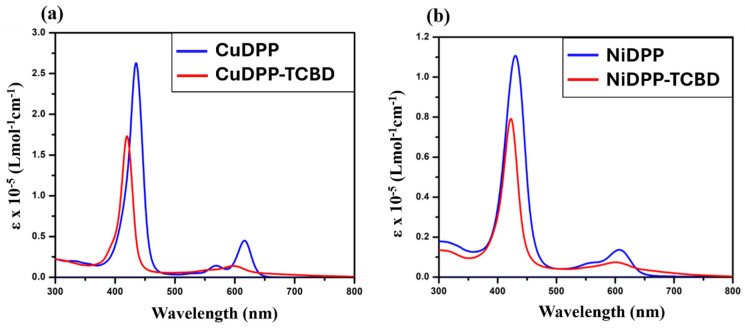
Absorption spectra of (**a**) **CuDPP** and **CuDPP-TCBD**, and (**b**) **NiDPP** and **NiDPP-TCBD** in CH_2_Cl_2_ at 298 K.

Details of the synthesis are provided in the experimental section, and Scheme 1 outlines the key steps. Briefly, (5-Bromo-15-formyl-10,20-diphenylporphyrinato) nickel(II) was synthesized according to the method reported by Ng and co-workers via formylation followed by bromination of diphenylporphyrin [27]. Copper metalation of 5-bromo-15-formyl-10,20-diphenylporphyrin was also subsequently performed. The push–pull *meso*-TCBD-functionalized diphenylporphyrins were synthesized via the [2 + 2] cycloaddition– retroelectrocyclization reaction shown in Scheme 1. Here, 5-bromo-15-formyl-10,20-diphenylporphyrin metal(II) (M = Ni, Cu) complexes were reacted in 1,4-dioxane with tributyl(phenylethynyl)stannate, resulting in the formation of diphenylporphyrins appended with a phenylethylformyl group. The reaction of TCNE with phenylethynyl-appended diphenylporphyrins in 1,2-dichloroethene for 2–3 h leads to the formation of a new class of donor–acceptor diphenylporphyrins that are substituted with TCBD groups. This process yields the desired compounds in 70–75% yield. Both **NiDPP-TCBD** and **CuDPP-TCBD** were analysed by ^1^H and ^13^C (Figure 1 and Figures S1–S4), UV-vis (Figure 2), MALDI-TOF-MS (Figures S5–S8), and electrochemistry (Figure 3).

Generally, [2 + 2] reactions occur between the electrically deficient dienophiles TCNE and TCNQ and the electrically rich diene alkynes coupled to electron-donor groups such as triphenylamine, thiophene, phenothiazine, and BODIPY, which undergo a cycloaddition–retroelectrocyclization process [28]. Notably, Gryko and co-workers have indicated that *p*-cyanophenyl or *p*-nitrophenyl containing *trans*-A2B2 porphyrins did not react with TCNE [19]. In our case, the *meso*-CHO group exerted a negligible electronic effect and did not significantly alter the reaction direction.

### 2.2. Single Crystal Structure

High-quality single crystals of **NiDPP** were grown using a CHCl_3_–hexane solution at room temperature. Figure 1 shows the presence of nickel in the porphyrin cavity. The crystal structure of **NiDPP** revealed a triclinic crystal system (space group = P − 1) with one molecule in the asymmetric unit of the unit cell. Table S1 provides the crystallographic data and selected bond lengths, while Figure 1 illustrates these aspects. It should be noted that during crystal structure refinement, the Ni atom exhibited disorder, necessitating modelling and constraints to achieve an accurate representation. The occupancy values for NiDPP, NiA, and NiB were determined to be 0.84, 0.08, and 0.08, respectively. Additionally, the displacements of the 24 core atoms (Δ24) and the *β*-pyrrole carbons (ΔC*_β_*) were measured at 0.289 Å and 0.363 Å, respectively. There is a C-H---π interaction between the *meso*-phenyl ring (C-H) and the π-cloud of the pyrrole ring with a distance of 2.88 Å. Further, a weak π–π interaction with a distance of 3.586 Å is observed between the *meso*-phenyl ring and the porphyrin π system.

### 2.3. NMR Characterisation

**NiDPP** and **NiDPP-TCBD** compounds were characterized using NMR spectroscopy in CDCl_3_ at 298 K, as shown in Figures S1–S4. NiDPP exhibited characterized signals for *meso*-formyl protons, phenyl protons, and *meso*-TCBD phenyl protons [18,19]. The comparative ^1^H NMR spectra of **NiDPP** and **NiDPP-TCBD** are shown in Figure 2. **NiDPP** exhibited one singlet for *meso*-CHO protons, four doublets for *β* protons, and a multiplet for phenyl and phenylethynyl 15 protons; however, after the introduction of TCBD at the *meso* position, the ^1^HNMR spectrum of **NiDPP-TCBD** was modulated. There is an upfield shift in the *meso*-TCBD-phenyl proton compared to **NiDPP**. There is a deshielding effect on all β protons due to the reduction in the ring current. Moreover, *meso*-TCBD-phenyl protons are upshielded compared to **NiDPP**.

### 2.4. Optical Absorption Studies

To explain the substitution effect at the *meso*-TCBD appended Ni and Cu diphenylporphyrins, absorption spectra were recorded in CH_2_Cl_2_ at 298 K, and are displayed in Figure 3, while the data are summarized in Table 1. **NiDPP** displayed the Soret band at 430 nm and Q bands at 555 and 607. Introduction of TCBD moieties at the *meso* position results in a blue shift in the Soret band (Δλ_max_ = 8 nm) for **NiDPP-TCBD** and (Δλ_max_ = 15 nm) for **CuDPP-TCBD**, the observable band indicating electronic interaction between entities. In both **NiDPP-TCBD** and **CuDPP-TCBD**, there were broad spectral features covering the 650–800 nm range attributable to intramolecular charge transfer [29,30].

### 2.5. Electrochemistry

**Figure 4:**
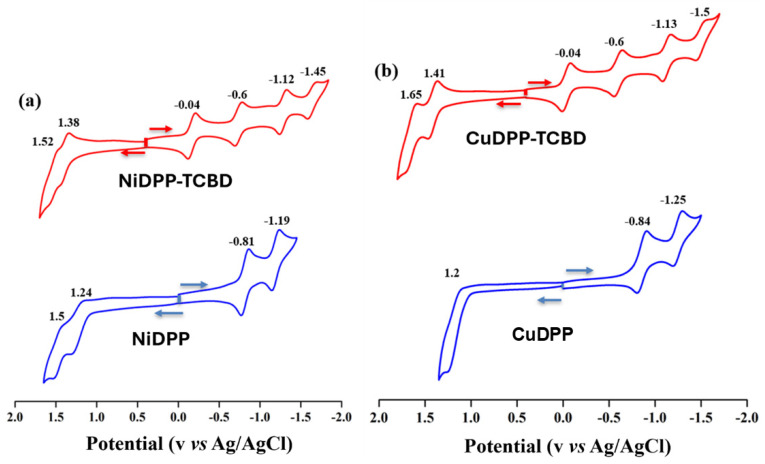
Cyclic voltammograms of (**a**) **NiDPP** and **Ni-TCBD** and (**b**) **CuDPP** and **Cu-TCBD** in CH_2_Cl_2_ containing 0.1 M TBAPF_6_ at 298 K (scan rate = 100 mV/s).

To elucidate the effect of formyl and TCBD moieties, electrochemical redox studies of synthesized porphyrins were investigated in dry CH_2_Cl_2_ containing 0.1 M TBAPF_6_ at 298 K. In general, porphyrins exhibit two reversible one-electron oxidations and two one-electron reductions [22]. **NiDPP** showed two reversible one-electron-ring oxidations at 1.24 and 1.50 V and two reversible one-electron-ring reductions at −0.81 and −1.19 V, while **CuDPP** showed only one quasi-reversible ring oxidation at 1.20 V and two reversible ring reductions at −0.84 and −1.25 V (Figure 4), which are anodically shifted as compared to MTTPs (M = Ni(II) and Cu(II)) [22]. The introduction of the TBCB unit at the *meso* position generated six redox processes, with two additional reductions attributable to the TCBD unit. The corresponding redox potentials of **NiDPP-TCBD** were located at 1.52, 1.38, −0.04, −0.60, −1.12, and −1.45 V vs. Ag/AgCl, while those of **CuDPP-TCBD** were located at 1.65, 1.41, −0.04, −0.60, −1.13, and −1.50 V (Table 2). Harder oxidation of MDPP-TCBD over MDPP (where M = Ni and Cu) for these *meso*-functionalized porphyrins was observed owing to the push–pull effect. The first-ring oxidations in MDPP-TCBD were 140–210 mV more positive than in MDPP (Figure 4). Interestingly, the first two reductions in MDPP-TCBD (M = Ni and Cu) correspond to the TCBD moiety [18]. Additionally, the third and fourth reductions correspond to the reduction of the porphyrin ring and are cathodically shifted due to the presence of TCBD [18].

### 2.6. DFT Studies

**Figure 5:**
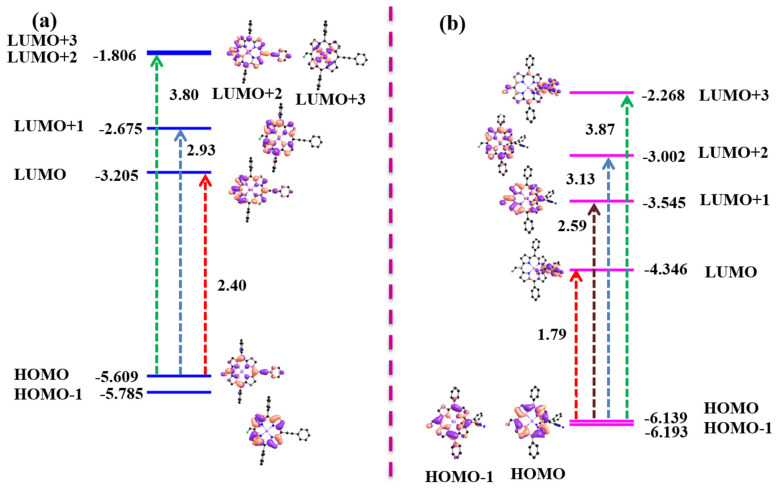
Frontier molecular orbitals profiles and energy diagram of (**a**) **NiDPP** and (**b**) **NiDPP-TCBD**.

To understand the effects of the PE and TCBD groups on structural properties, the ground-state geometries of the synthesized porphyrins were optimized using B3LYP/LANL2DZ in the gas phase. **NiDPP** and **NiDPP-TCBD** exhibit ruffled conformation, as shown in Figure 5. **NiDPP-TCBD** (Δ24 = 0.287 Å and ΔCβ = 0.226 Å) is more nonplanar than NiDPP (Δ24 = 0.264 Å and ΔCβ = 0.219 Å). In **NiDPP-TCBD**, nickel metal is located 0.010 Å above the plane, while it is 0.016 Å in **NiDPP**. The copper PE- and TCBD-appended porphyrins (**CuDPP** and **CuDPP-TCBD**) are relatively much more planar than the corresponding nickel porphyrins. The porphyrin ring is almost planar, having Δ24 = 0.042 Å and ΔC*_β_* = 0.033 Å for **Cu-1** and Δ24 = 0.108 Å and ΔC*_β_* = 0.107 Å for **Cu-2**.

The frontier molecular orbitals (FMOs) in *meso*-PE-substituted complexes (**NiDPP** and **CuDPP**) exhibited electron density localized on the diphenylporphyrin (DPP) ring and *meso* substituents, as shown in Figure 5a and Figure S9a. The HOMO and LUMO energy gaps were found to be in the range of 2.40–2.22 eV. The electron density of HOMO-1 and HOMO was localized on the DPP in **NiDPP-TCBD** and **CuDPP-TCBD**, as shown in Figure 5b and Figure S9b. However, the electron densities in the LUMO and LUMO + 3 are localized on the TCBD substituent. However, the electron densities in LUMO + 1 and LUMO + 2 were localized mainly on the DPP ring. The HOMO and LUMO energy gap in *meso*-TCBD-appended porphyrins (**NiDPP-TCBD** and **CuDPP-TCBD**) was found to be in the range of 1.87–1.79 eV, which is lower than that of the *meso*-PE-appended porphyrins (**NiDPP** and **CuDPP**) due to extensive stabilization of HOMO and LUMO. The localization of electron density from DPP to TCBD substituent indicates charge transfer interactions between TCBD and the DPP ring.

### 2.7. Spectroelectrochemical Studies

**Figure 6:**
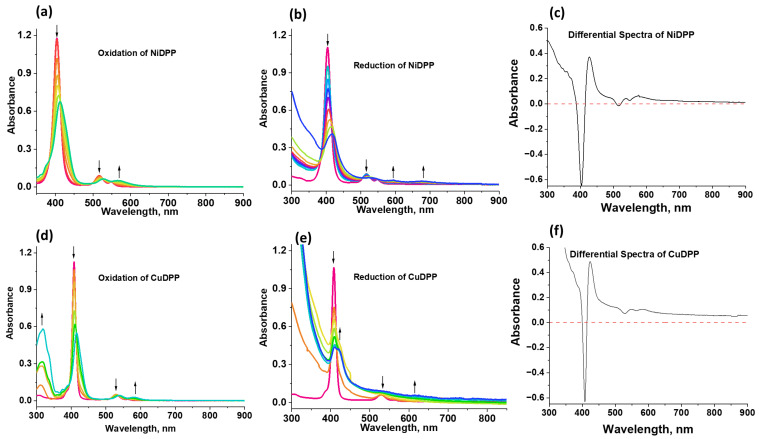
Spectroelectrochemical changes during the first oxidation (**a**,**d**) and the first reduction (**b**,**e**) of NiDPP and CuDPP in the solvent benzonitrile (PhCN). (**c**,**f**) represent the spectrum deduced for the charge-separated state (see text for details).

Spectroelectrochemical experiments were performed on NiDPP and CuDPP in benzonitrile to identify the peaks corresponding to cationic and anionic species (Figure 6). In both NiDPP and CuDPP, cationic peak formation is clearly observed at around 570 nm and 580 nm, respectively (see Figure 6a,d). Similarly, anionic peaks for NiDPP were observed at 595 nm and 685 nm (Figure 6b), whereas for CuDPP, a cationic peak was observed at 635 nm (Figure 6e). All these processes were reversible, except for CuDPP, for which about 95% of the original intensity could be recovered. To get a clear view of the cationic and anionic peaks, their corresponding differential spectra (sum of cationic and anion spectra minus that of the neutral compound) are also shown in Figure 6c,f.

### 2.8. Energy Level Diagram and Femtosecond Transient Absorption Spectral Studies

**Figure 7:**
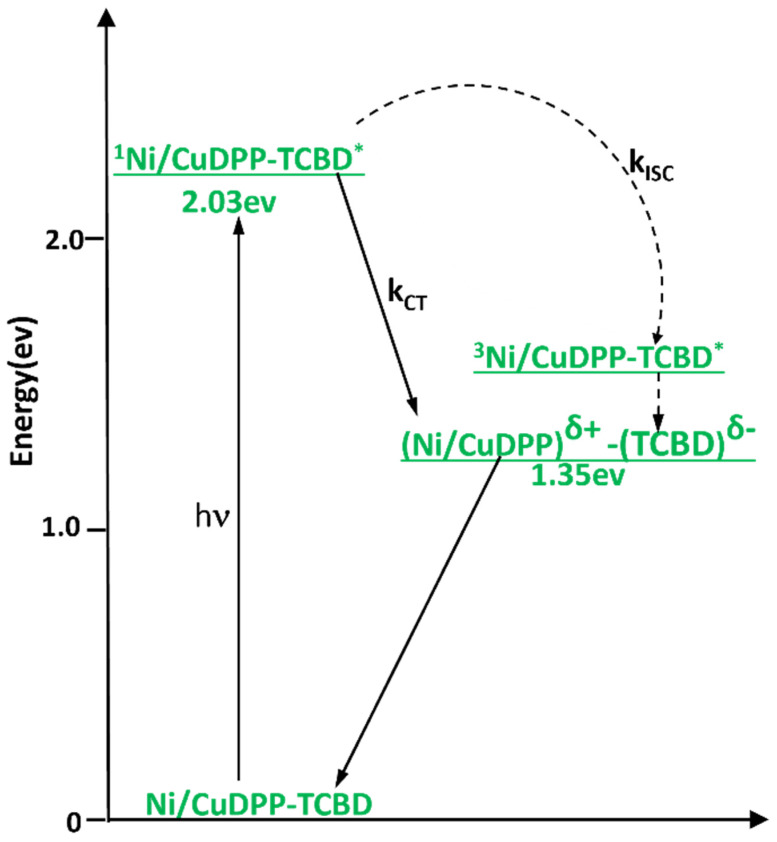
Energy level diagram in the solvent PhCN. (Solid arrows indicate major photo-processes, dashed arrows indicate minor photo-processes).

Energy level diagrams of **NiDPP-TCBD** and **CuDPP-TCBD** conjugates are shown in Figure 7 in solvent PhCN. It is clear that the energy of the charge-transfer state (MDPP^δ+^-TCBD^δ−^; M = Cu or Ni) is lower than that of the porphyrin triplet state, making the CT state thermodynamically more favorable than the triplet state via intersystem crossing. To obtain direct evidence of excited-state events upon photoexcitation, femtosecond transient absorption experiments were performed in the solvent benzonitrile (PhCN) using excitation at the Soret band (around 420 nm).

**Figure 8:**
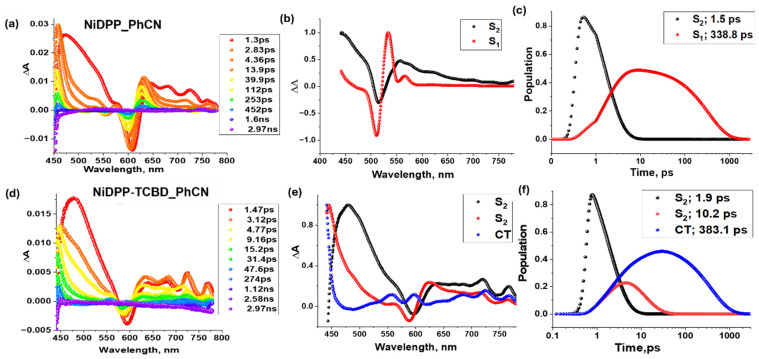
Femtosecond transient absorption spectra at indicated delay times (λ_ex_ = 420 nm) of (**a**) **NiDPP** and (**d**) **NiDPP-TCBD** in the solvent benzonitrile (PhCN). In the middle panel, their respective decay-associated spectra (**b**,**e**) and their population curves (**c**,**f**) from GloTarAn are shown in the right panel with the lifetimes of each excited state.

Figure 8 shows the femtosecond transient absorption spectra at the indicated delay times for **NiDPP** and **NiDPP-TCBD**, along with their decay-associated spectrum (DAS) and population curves on their right. A significant difference is evident in the spectral features (see Figure 8a,d). In early time-scale spectra of **NiDPP**, the instantaneously formed singlet excited state showed excited-state absorption (ESA) peaks at 455, 630, and 715 nm. In addition, negative peaks at 555 nm and 610 nm correspond to ground-state bleach (GSB), which was also observed. The decay of positive peaks and recovery of negative peaks were accompanied by the slow growth of a new transient peak around 530 nm (see Figure 8a), assigned to the population of the ^3^NiDPP* state formed via intersystem crossing (ISC), while in the case of **NiDPP-TCBD**, ESA peaks were observed at 460, 635, 680, 715, and 770 nm. An additional negative peak was also observed around 595 nm due to the GSB. By comparing these spectra with the spectroelectrochemical data shown in Figure 6, the newly formed peaks at 550 nm and 685 nm correspond to the cationic and anionic species of **NiDPP-TCBD**, respectively, confirming the population of the charge-transfer (CT) state rather than the triplet state. Here, the CT state population is promoted by the strong electron acceptor group TCBD, which was absent from the other two nickel compounds reported here. To obtain the precise lifetimes of each excited state and the population curves, target analysis was performed using the software GloTarAn 1.5.1 [42], as shown in the two right columns of Figure 8. Target analysis of **NiDPP** follows a two-component decay scheme corresponding to the S_2_ and S_1_ excited states. However, **NiDPP-TCBD** undergoes a three-component decay, with the CT state being thermodynamically more favorable (see Figure 7 for the energy-level diagram).

**Figure 9:**
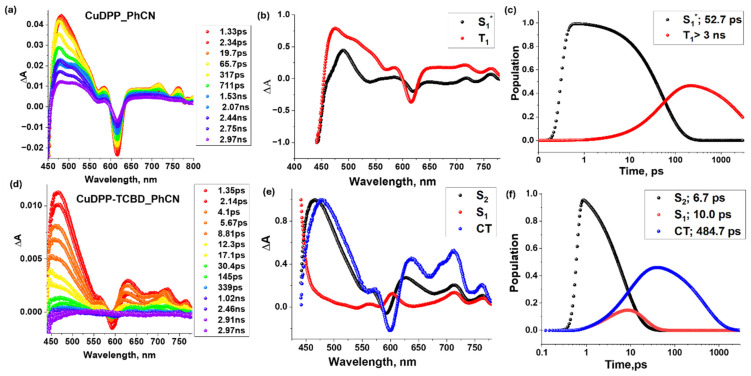
Femtosecond transient absorption spectra at indicated delay times (λ_ex_ = 420 nm) of (**a**) **CuDPP** and (**d**) **CuDPP-TCBD** in the solvent benzonitrile (PhCN). In the middle panel, their respective decay-associated spectra (**b**,**e**) and their population kinetic curves (**c**,**f**) are shown in the right panel with the lifetimes of each excited state.

Femtosecond transient absorption spectra of **CuDPP** and **CuDPP-TCBD** and their target analysis data are shown in Figure 9 in solvent PhCN with excitation wavelength at their Soret band around 420 nm. The ESA peaks of **CuDPP** were observed at 475, 585, 715, and 765 nm (see Figure 9a), with a negative peak at 620 nm that is assigned to GSB. As the positive peaks decayed and the negative peaks recovered, a new peak emerged at 512 nm, corresponding to either the ring-centered triplet-state population of ^3^CuDPP* or the metal-centered ^2^[d_z2_, d_x2-y2_] state [43]. The lifetime of this triplet state is beyond the limit of the delay line of our instrument, that is, 3 ns. In the case of **CuDPP-TCBD**, ESA peaks were observed at 465, 625, and 720 nm, accompanied by a negative peak at 590 nm corresponding to GSB. Spectro-electrochemical data shown in Figure 6 confirm that the peaks at 570 nm and 635 nm correspond to the intermediate charge-transfer state. The target analysis shown to the right of the corresponding spectra in Figure 9 provides information on the lifetimes of these excited states and their populations over time. Target analysis of **CuDPP-TCBD** follows a three-component decay, with the CT state having a lifetime of 484.7 ps.

The target analysis indicates that the triplet or copper-d^9^-originated doublet state was significantly populated only in CuDPP, whereas in CuDPP-TCBD, it was either not populated or decayed too rapidly to be detected by the target analysis. In CuDPP-TCBD, the decay proceeded to the CT state of **CuDPP-TCBD** with a lifetime of 484.7 ps.

## 3. Experimental Section

### 3.1. Materials

Tributyl(phenylethynyl)tin was purchased from Sigma-Aldrich (St. Luis, MO, USA). Cu(OAc)_2_•H_2_O, Ni(OAc)_2_, and benzaldehyde were purchased from HiMedia (Kelton, PA, USA). Pyrrole and tetracyanoethylene (TCNE) were purchased from Alfa Aesar (Haverhill, MA, USA).

### 3.2. Instrumentation and Methods

The photophysical data were measured using a Shimadzu UV–1800 spectrophotometer (Columbia, MD, USA) with a pair of 3.5 cm^3^ cuvettes and a 10 mm path length. Triple-distilled dichloromethane (DCM) was used for electrochemical studies. The ^1^H and ^13^C NMR spectra were recorded on JEOL ECX 400 MHz (Peabody, MA, USA) and 125 MHz spectrometers, respectively, using CDCl_3_ as the solvent at 298 K. MALDI-TOF MS were recorded on a Bruker UltrafleXtreme-TN MALDI-TOF spectrometer (Billerica, MA, USA), at 298 K. Cyclic voltammetric studies were carried out in A CHI 620E work station (Austin, TX, USA) with a three-electrode assembly consisting of a platinum electrode working electrode, a platinum counter electrode, and a saturated Ag/AgCl reference electrode at 298 K under argon atmosphere in CH_2_Cl_2_ at 298 K.

### 3.3. Femtosecond Laser Flash Photolysis

Femtosecond transient studies were performed using an Ultrafast Femtosecond Laser Source (Astrella) by Coherent (Saxonburg, PA, USA), which incorporates a diode, mode-locked Ti:Sapphire laser (Vitara) and a diode-pumped intracavity-doubled Nd:YLF laser (Revolution) to generate a fundamental compressed laser of 800 nm and power output of 5.24 W. A Helios Transient Absorption Spectrometer coupled with a Femtosecond Harmonics Generator, both provided by Ultrafast Systems LLC (Sarasota, FL, USA), will be used for optical detection. The source for the pump pulse was derived from the fundamental output of Astrella (compressed output 5.24 W, pulse width 100 fs, 800 nm at a repetition rate of 1 kHz) by introducing 95% of the beam into the OPA, while the other 5% was sent to the delay line and white-light-generating crystal. The beam passing through the OPA is called the pump beam, as it excites the sample. The beam transmitted through the delay line and crystal is termed the probe beam, as it reveals the spectral changes in the sample over time. The OPA takes the 800 nm fundamental and converts it to a specific wavelength in the 400–2200 nm range, allowing the excitation wavelength to be selected. Kinetic traces at appropriate wavelengths were assembled from the time-resolved spectral data. Data analysis was performed using GloTarAn 1.5.1 and Surface Xplorer 4.5.14 software. All measurements were conducted in degassed solutions at 298 K.

### 3.4. Synthesis

The *meso*-phenylacetylene carrying MDPPs (M = Ni and Cu; see Chart 1 for structures) were synthesized by reacting MDPPCHO(Br) (30 mg, 48.08 mmol) and tributyl(phenyl-ethynyl)stannate (Sigma-Aldrich, St. Louis, MO, USA) (1 equivalent) with Pd(PPh_3_)_4_ (20 mol%) in 25 mL dry 1,4-dioxane for 4 h. UV-vis and TLC were used to monitor the reaction’s progress. At the end, the reaction mixture was cooled, and the solvent was removed under reduced pressure. The crude was purified by column chromatography on silica gel (100–200 mesh) using a 4:1 CHCl_3_/hexane (*v*/*v*) mixture as the eluent.

**NiDPP**: Black solid (yield: 25 mg, 0.034 mmol, 80%). UV–vis (nm, log ε): 430 (5.04), 555 (3.84), 607 (4.13). ^1^H NMR (500 MHz, CDCl_3_): *δ* (ppm) 12.04 (s, 1H, *meso*-H), 9.76–9.75 (d, 2H, *β*-H), 9.49–9.48 (d, 2H, *β*-H), 8.81–8.79 (d, 2H, *β*-H), 8.67–8.66 (d, 2H, *β*-H), 7.97–7.92 (m, 6H, Ph-H), 7.74–7.70 (m, 6H, Ph-H/*meso*-Ph-H), 7.54–7.49 (m, 3H, *meso*-Ph-H). ^13^C NMR (125 MHz, CDCl_3_) *δ* (ppm): 192.52, 144.65, 143.66, 141.46, 139.92, 135.43, 133.43, 132.82, 132.23, 130.96, 129.31, 129.09, 128.41, 127.78, 123.26, 121.72, 115.31, 107.36, 104.12, 98.40, 89.76, 30.17. MS (MALDI-TOF) *m*/*z*: [M + H]^+^ found 647.299, calctd. 647.138. Elem. anal. calctd (%) for C_41_H_24_N_4_NiO: C, 76.07; H, 3.74; N, 9.07; found: C, 75.89; H, 3.59; N, 8.92.

**CuDPP**: Black solid (yield: 26 mg, 0.039 mmol, 84%). MS (MALDI-TOF) *m*/*z*: [M]^+^ found 651.260, calctd. 651.125. Elem. anal. calctd (%) for C_41_H_24_CuN_4_O: C, 75.50; H, 3.71; N, 8.59; found: C, 75.21; H, 3.59; N, 8.42. Note—Due to the paramagnetic nature of CuDPP, no NMR could be obtained.

**MDPP-TCBD:** MDPP (20 mg, 0.031 mmol) and TCNE (2.0 equivalents) were dissolved in 1,2-dichloroethane and refluxed for 3 h. Reaction progress was monitored by TLC and UV-vis spectroscopy. The reaction was allowed to cool to ambient, and the solvent was removed under reduced pressure. Crude was purified by silica (100–200 mesh) column chromatography using a 7:3 CHCl_3_/hexane (*v*/*v*) mixture as the eluent.

**NiDPP-TCBD**: Black solid (yield: 17 mg, 0.018 mmol, 70%). UV–vis (nm, log ε): 422 (4.89), 600 (3.86). ^1^H NMR (500 MHz, CDCl_3_): *δ* (ppm) 12.06 (s, 1H, *meso*-H), 9.81–9.80 (d, 2H, *β*-H), 8.88–8.85 (m, 4H, *β*-H), 8.77–8.76 (d, 2H, *β*-H), 7.94–7.92(d, 4H, *o*-Ph-H), 7.77–7.70 (m, 6H-*m-p-*Ph), 7.38–7.24 (m, 5H-TCBD-Ph). ^13^C NMR (125 MHz, CDCl_3_) *δ* (ppm): 193.15, 168.43, 165.67, 144.03, 143.40, 139.70, 139.12, 136.02, 135.73, 134.78, 132.90, 129.81, 128.60, 127.36, 121.83, 115.60, 112.20, 109.44, 107.61, 98.30, 92.76, 30.06. MS (MALDI-TOF) *m*/*z*: [M]^+^ found 774.305, calctd. 774.14. Elem. anal. calcd (%) for C_47_H_24_N_8_NiO: C, 72.80; H, 3.12; N, 14.45; found: C, 72.65; H, 2.98; N, 14.21.

**CuDPP-TCBD**: Black solid (yield: 18 mg, 0.020 mmol, 75%). MS (MALDI-TOF) *m*/*z*: [M]^+^ found 779.251, calctd. 779.14. Elem. anal. calcd (%) for C_47_H_24_CuN_8_O: C, 72.35; H, 3.10; N, 14.36; found: C, 72.11; H, 3.01; N, 14.18. Note—Due to the paramagnetic nature of CuDPP, no NMR could be recorded.

## 4. Summary

Push–pull systems containing Ni and Cu at the porphyrin cavity and TCBD at the *meso* position have been newly synthesized and characterized using a range of physicochemical techniques. The X-ray structure of one of the precursor molecules, NiDPP, was also solved. Multiple redox processes were observed for these compounds corresponding to both MDPP and TCBD entities, most of which were electrochemically reversible. Spectroelectrochemical studies of the first oxidation and first reduction processes were used to deduce the transient spectral characteristics of the charge-transfer process. Frontier orbitals derived from DFT calculations were used to assess the donor and acceptor sites in push–pull systems. HOMO on the MDPP and LUMO on TCBD, revealing their roles as electron donor and electron acceptor, was also supported by the electrochemical and spectroelectrochemical studies. From the energy diagram, intramolecular charge transfer was found to be feasible in both MDPP-TCBD systems. Further pump–probe spectroscopy helped to characterize the charge-transfer states. Lifetimes of 383 ps and 485 ps were obtained for NiDPP-TCBD and CuDPP-TCBD in benzonitrile, respectively, highlighting their potential for light-energy harvesting and other optoelectronic applications.

## Figures and Tables

**Table 1:** Optical absorption data for the investigated compounds in CH_2_Cl_2_ at 298 K.

Porphyrins	λ_max,_ nm (log ε)	
	Soret Band	Q Band
CuDPP	435 (5.41)	568 (4.14), 615 (4.65)
CuDPP-TCBD	420 (5.23)	558 (3.96), 601 (4.13)
NiDPP	430 (5.04)	555 (3.84), 607 (4.13)
NiDPP-TCBD	422 (4.89)	600 (3.86)

**Table 2:** Redox potential data of investigated porphyrins in CH_2_Cl_2_ containing 0.1 M TBAPF_6_ at 298 K.

Complex	Oxidation (V vs. Ag/AgCl)		Reduction (V vs. Ag/AgCl)			
	I	II	I	II	III	IV
NiDPP	1.24	1.50	−0.81	−1.19	-	-
NiDPP-TCBD	1.38	1.52	−0.04	−0.60	−1.12	−1.45
CuDPP	1.20	-	−0.84	−1.25	-	-
CuDPP-TCBD	1.41	1.65	−0.04	−0.60	−1.13	−1.50

## Data Availability

Data are contained within the article and Supplementary Materials.

## Supplementary Materials

The following supporting information can be downloaded at: https://www.mdpi.com/article/10.3390/molecules31060934/s1. Figure S1. ^1^H NMR spectrum of NiDPP in CDCl_3_ at 298 K. Figure S2. ^1^H NMR spectrum of NiDPP-TCBD in CDCl_3_ at 298 K. Figure S3. ^13^C NMR spectrum of NiDPP in CDCl_3_ at 298 K. Figure S4. ^13^C NMR spectrum of NiDPP-TCBD in CDCl_3_ at 298 K. Figure S5. MALDI-TOF Mass spectrum of NiDPP in CH_2_Cl_2_ at 298 K. Figure S5A. Simulated (bottom) and observed (top) MALDI TOF mass spectra of expanded NiDPP molecular ion peak. Figure S6. MALDI-TOF Mass spectrum of NiDPP-TCBD in CH_2_Cl_2_ at 298 K. Figure S6A. Simulated (bottom) and observed (top) MALDI TOF mass spectra of expanded NiDPP-TCBD molecular ion peak. Figure S7. MALDI-TOF mass spectrum of CuDPPin CH_2_Cl_2_ at 298 K. Figure S7A. Simulated (bottom) and observed (top) MALDI TOF mass spectra of expanded CuDPP molecular ion peak. Figure S8. MALDI-TOF Mass spectrum of CuDPP-TCBD in CH_2_Cl_2_ at 298 K. Figure S8A. Simulated (bottom) and observed (top) MALDI TOF mass spectra of expanded CuDPP-TCBD molecular ion peak. Figure S9. Optimized geometries showing top as well as side views of (a) CuDPP and (b) CuDPP-TCBD. Figure S10. Deviation of the porphyrin 24-core atoms from the mean plane for (a) NiDPP-TCBD and (b) CuDPP-TCBD. Table S1. Selected bond distances and crystallographic data for NiDPP. Table S2. Selected bond lengths (Å) and bond angles (º) for the B3LYP/LANL2DZ optimised geometries of MTPPand MTPP-TCBD (M = Ni and Cu).

## References

[1] Sekita M., Ballesteros B., Diederich F., Guldi D.M., Bottari G., Torres T. (2016). Intense Ground-State Charge-Transfer Interactions in Low-Bandgap, Panchromatic Phthalocyanine–Tetracyanobuta-1,3-diene Conjugates. Angew. Chem..

[2] Milan K.A., Corinne Boudon B., Jean-Paul Gisselbrecht B., Barbara Enko C., Paul Seiler A., Imke B., Nicolle Langer D., Peter D., Jarowski D., Georg Gescheidt A. (2009). Organic Super-Acceptors with Efficient Intramolecular Charge-Transfer Interactions by [2 + 2] Cycloadditions of TCNE, TCNQ, and F_4_-TCNQ to Donor-Substituted Cyanoalkynes. Chem.-A Eur. J..

[3] Michinobu T., Diederich F. (2018). The [2 + 2] Cycloaddition-Retroelectrocyclization (CA-RE) Click Reaction: Facile Access to Molecular and Polymeric Push-Pull Chromophores. Angew. Chem. Int. Ed. Engl..

[4] Kivala M., Boudon C., Gisselbrecht J.-P., Seiler P., Gross M., Diederich F., Kivala M., Seiler P., Diederich F., Boudon C. (2007). Charge-Transfer Chromophores by Cycloaddition–Retro-Electrocyclization: Multivalent Systems and Cascade Reactions. Angew. Chem. Int. Ed..

[5] Zang X., Liu H., Li Q., Li Z., Li Z. (2020). A TCBD-Based AB_2_-Type Second-Order Nonlinear Optical Hyperbranched Polymer Prepared by a Facile Click-Type Postfunctionalization. Polym. Chem..

[6] Kato S.I., Diederich F. (2010). Non-Planar Push–Pull Chromophores. Chem. Commun..

[7] Bui A.T., Philippe C., Beau M., Richy N., Cordier M., Roisnel T., Lemiègre L., Mongin O., Paul F., Trolez Y. (2020). Synthesis, Characterization and Unusual near-Infrared Luminescence of 1,1,4,4-Tetracyanobutadiene Derivatives. Chem. Commun..

[8] Rout Y., Mobin S.M., Misra R. (2019). Tetracyanobutadiene (TCBD) Functionalized Benzothiadiazole Derivatives: Effect of Donor Strength on the [2 + 2] Cycloaddition–Retroelectrocyclization Reaction. New J. Chem..

[9] Lapidus S.H., Stephens P.W., Fumanal M., Ribas-Ariño J., Novoa J.J., Dasilva J.G., Rheingold A.L., Miller J.S. (2021). Low Temperature Structures and Magnetic Interactions in the Organic-Based Ferromagnetic and Metamagnetic Polymorphs of Decamethylferrocenium 7,7,8,8-Tetracyano-p-Quinodimethanide, [FeCp*2]˙^+^[TCNQ]˙^−^. Dalton Trans..

[10] Cheung H.F.H., Chilcote M., Yusuf H., Cormode D.S., Shi Y., Kurfman S., Franson A., Flatté M.E., Johnston-Halperin E., Fuchs G.D. (2021). Raman Spectroscopy and Aging of the Low-Loss Ferrimagnet Vanadium Tetracyanoethylene. J. Phys. Chem. C.

[11] Her J.H., Stephens P.W., Davidson R.A., Min K.S., Bagnato J.D., Van Schooten K., Boehme C., Miller J.S. (2013). Weak Ferromagnetic Ordering of the Li^+^[Tcne]^•−^ (Tcne = Tetracyanoethylene) Organic Magnet with an Interpenetrating Diamondoid Structure. J. Am. Chem. Soc..

[12] Kivrak A., Zobi C., Torlak Y., Çamlısoy Y., Kuş M., Kivrak H. (2018). Synthesis of Tetracyanoethylene-Substituted Ferrocene and Its Device Properties. Appl. Organomet. Chem..

[13] Fang Y., Koszelewski D., Kadish K.M., Gryko D.T. (2014). Facile Electrosynthesis of π-Extended Porphyrins. Chem. Commun..

[14] Esipova T.V., Vinogradov S.A. (2014). Synthesis of Phosphorescent Asymmetrically π-Extended Porphyrins for Two-Photon Applications. J. Org. Chem..

[15] Berna B., Nardis S., Galloni P., Savoldelli A., Stefanelli M., Fronczek F.R., Smith K.M., Paolesse R. (2016). β-Pyrrolopyrazino Annulated Corroles via a Pictet-Spengler Approach. Org. Lett..

[16] Yadav I., Shanu M., Acharyya J.N., Prakash G.V., Sankar M. (2022). Ultrafast Dynamics and Strong Two-Photon Absorption Properties of Nonplanar β-Functionalized “Push-Pull” Copper Corroles with a Mixed Substituent Pattern. Inorg. Chem..

[17] Paolesse R., Nardis S., Monti D., Stefanelli M., Di Natale C. (2017). Porphyrinoids for Chemical Sensor Applications. Chem. Rev..

[18] Rohal R.K., Acharyya J.N., Shanu M., Prakash G.V., Sankar M. (2022). β-Tetracyanobutadiene-Appended Porphyrins: Facile Synthesis, Spectral and Electrochemical Redox Properties, and Their Utilization as Excellent Optical Limiters. Inorg. Chem..

[19] Koszelewski D., Nowak-Król A., Gryko D.T. (2012). Selective Cycloaddition of Tetracyanoethene (TCNE) and 7,7,8,8-Tetracyano-p-Quinodimethane (TCNQ) to Afford *meso*-Substituted Phenylethynyl Porphyrins. Chem. Asian J..

[20] Liu X., Wang D., Gao H., Yang Z., Xing Y., Cao H., He W., Wang H., Gu J., Hu H. (2016). Nonlinear Optical Properties of Symmetrical and Asymmetrical Porphyrin Derivatives with Click Chemistry Modification. Dye. Pigm..

[21] Liang P., Du Z., Wang D., Yang Z., Sheng H., Liang S., Cao H., He W., Yang H. (2014). Optoelectronic and Self-Assembly Properties of Porphyrin Derivatives with Click Chemistry Modification. ChemPhysChem.

[22] Sekaran B., Dawson A., Jang Y., MohanSingh K.V., Misra R., D’Souza F. (2021). Charge-Transfer in Panchromatic Porphyrin-Tetracyanobuta-1,3-Diene-Donor Conjugates: Switching the Role of Porphyrin in the Charge Separation Process. Chem.-A Eur. J..

[23] Yadav I., Sharma J.K., Sankar M., D’Souza F. (2023). Symmetrically Functionalized Copper and Silver Corrole-Bis-Tetracyanobutadiene Push-Pull Conjugates: Efficient Population of Triplet States via Charge Transfer. Chem.-A Eur. J..

[24] Huang T.H., Chen Y.J., Lo S.S., Yen W.N., Mai C.L., Kuo M.C., Yeh C.Y. (2006). Highly Conjugated Multiporphyrins: Synthesis, Spectroscopic and Electrochemical Properties. Dalton Trans..

[25] Drobizhev M., Meng F., Rebane A., Stepanenko Y., Nickel E., Spangler C.W. (2006). Strong Two-Photon Absorption in New Asymmetrically Substituted Porphyrins: Interference between Charge-Transfer and Intermediate-Resonance Pathways. J. Phys. Chem. B.

[26] Senge M.O., Fazekas M., Pintea M., Zawadzka M., Blau W.J. (2011). 5,15-A_2_B_2_- and 5,15-A_2_BC-Type Porphyrins with Donor and Acceptor Groups for Use in Nonlinear Optics and Photodynamic Therapy. Eur. J. Org. Chem..

[27] Yeung M., Ng A.C.H., Drew M.G.B., Vorpagel E., Breitung E.M., McMahon R.J., Ng D.K.P. (1998). Facile Synthesis and Nonlinear Optical Properties of Push-Pull 5,15 -Diphenylporphyrins. J. Org. Chem..

[28] Rout Y., Gautam P., Misra R. (2017). Unsymmetrical and Symmetrical Push-Pull Phenothiazines. J. Org. Chem..

[29] Wu Y.L., Stuparu M.C., Boudon C., Gisselbrecht J.P., Schweizer W.B., Baldridge K.K., Siegel J.S., Diederich F. (2012). Structural, Optical, and Electrochemical Properties of Three-Dimensional Push-Pull Corannulenes. J. Org. Chem..

[30] Winterfeld K.A., Lavarda G., Guilleme J., Guldi D.M., Torres T., Bottari G. (2019). Subphthalocyanine–Tetracyanobuta-1,3-Diene–Aniline Conjugates: Stereoisomerism and Photophysical Properties. Chem. Sci..

[31] Peng X., Han J., Li X., Liu G., Xu Y., Peng Y., Nie S., Li W., Li X., Chen Z. (2023). Electrocatalytic hydrogen evolution with a copper porphyrin bearing *meso*-(o-carborane) substituents. Chem. Comm..

[32] Bansal D., Cardenas-Morcoso D., Boscher N. (2023). Conjugated porphyrin polymer films with nickel single sites for the electrocatalytic oxygen evolution reaction. J. Mater. Chem. A..

[33] Bansal D., Ghahramanzadehasl H., Cardenas-Morcoso D., Desport J., Frache G., Bengasi G., Boscher N.D. (2024). Directly-Fused Ni (II) Porphyrin Conjugated Polymers with Blocked *meso*-Positions: Impact on Electrocatalytic Properties. Chem.-A Eur. J..

[34] Tancini F., Monti F., Howes K., Belbakra A., Listorti A., Schweizer W.B., Reutenauer P., Alonso-Gómez J., Chiorboli C., Urner L.M. (2014). Cyanobuta-1, 3-dienes as Novel Electron Acceptors for Photoactive Multicomponent Systems. Chem.-A Eur. J..

[35] Philippe C., Bui A.T., Beau M., Bloux H., Riobé F., Mongin O., Roisnel T., Cordier M., Paul F., Lemiègre L. (2022). Synthesis and Photophysical Properties of 1, 1, 4, 4-Tetracyanobutadienes Derived from Ynamides Bearing Fluorophores. Chem.-A Eur. J..

[36] Yamada M., Rivera-Fuentes P., Schweizer W.B., Diederich F. (2010). Optical Stability of Axially Chiral Push–Pull-Substituted Buta-1, 3-dienes: Effect of a Single Methyl Group on the C60 Surface. Angew. Chem. Int. Ed..

[37] Marques P.S., Castán J.M.A., Raul B.A., Londi G., Ramirez I., Pshenichnikov M.S., Beljonne D., Walzer K., Blais M., Allain M. (2020). Triphenylamine/Tetracyanobutadiene-Based π-Conjugated Push–Pull Molecules End-Capped with Arene Platforms: Synthesis, Photophysics, and Photovoltaic Response. Chem.-A Eur. J..

[38] Winterfeld K.A., Lavarda G., Guilleme J., Sekita M., Guldi D.M., Torres T., Bottari G. (2017). Subphthalocyanines axially substituted with a tetracyanobuta-1,3-diene–aniline moiety: Synthesis, structure, and physicochemical properties. J. Am. Chem. Soc..

[39] Michinobu T., May J.C., Lim J.H., Boudon C., Gisselbrecht J., Seiler P., Gross M., Biaggio I., Diederich F. (2005). A new class of organic donor–acceptor molecules with large third-order optical nonlinearities. Chem. Commun..

[40] Kivala M., Diederich F. (2009). Acetylene-derived strong organic acceptors for planar and nonplanar push− pull chromophores. Acc. Chem. Res..

[41] Gupta P.K., Ileperuma C.V., Misra R., D’Souza F. (2025). Strong Acceptor Incorporated Phenothiazine-C_60_ Multi-Redox Push–pull Conjugates: Demonstration of C_60_’s Superior Electron Acceptor Characteristics. Chem. Sci..

[42] Snellenburg J.J., Laptenok S., Seger R., Mullen K.M., Van Stok-kum I.H. (2012). Glotaran: A Java-based graphical user interface for the R package TIMP. J. Stat. Software.

[43] Jeong D., Kang D.-g., Joo T., Kim S.K. (2017). Femtosecond-resolved state relaxation dynamics of Copper(II) tetraphenylporphyrin (CuTPP) after Soret band excitation. Sci. Rep..

