# Supplementary material for: Synthesis and Intramolecular Charge Transfer Studies on meso-Tetracyanobutadine-Functionalized Diphenylporphyrin Complexes Incorporating Copper and Nickel Metals
Source: Molecules. 2026 Mar 11;31(6):934. doi: 10.3390/molecules31060934 (PMC13029222; doi:10.3390/molecules31060934)
Supplement: Supplementary file 1 [file molecules-31-00934-s001.zip › molecules-4117298-supplementary.pdf]

Supporting Information

Synthesis and Intramolecular Charge Transfer Studies on *meso*-Tetracyanobutadine Functionalized Diphenylporphyrin Complexes Incorporating Copper and Nickel Metals

Sumit Kumar Yadav,[a] Jatan K. Sharma,[b] Muniappan Sankar[a] Francis D'Souza[*b]*

[a]Department of Chemistry, Indian Institute of Technology Roorkee, Roorkee 247667, India

Email: m.sankar@cy.iitr.ac.in

[b]Department of Chemistry, University of North Texas, 1155 Union Circle, #305070, Denton, TX 76203-5017 (USA)

E-mail: francis.dsouza@unt.edu

Table of Contents:

	Page No.
**Figures S1–S2** ^{1}H NMR spectrum of **NiDPP** and **NiDPP-TCBD** in $CDCl_3$ at 298 K.	S3
**Figures S3–S4** ^{13}C NMR spectra of **NiDPP** and **NiDPP-TCBD** in $CDCl_3$ at 298 K.	S4
**Figure S5.** MALDI-TOF Mass spectrum of **NiDPP** in CH_2Cl_2 at 298 K.	S5
**Figure S5A**. Simulated (bottom) and observed (top) MALDI TOF mass spectra of expanded **NiDPP** molecular ion peak.	S5
**Figure S6.** MALDI-TOF Mass spectrum of **NiDPP-TCBD** in CH_2Cl_2 at 298 K.	S6
**Figure S6A**. Simulated (bottom) and observed (top) MALDI TOF mass spectra of expanded **NiDPP-TCBD** molecular ion peak.	S6
**Figure S7.** MALDI-TOF mass spectrum of **CuDPP**in CH_2Cl_2 at 298 K.	S7
**Figure S7A**. Simulated (bottom) and observed (top) MALDI TOF mass spectra of expanded **CuDPP** molecular ion peak.	S7
**Figure S8.** MALDI-TOF Mass spectrum of **CuDPP-TCBD** in CH_2Cl_2 at 298 K.	S8
**Figure S8A**. Simulated (bottom) and observed (top) MALDI TOF mass spectra of expanded **CuDPP-TCBD** molecular ion peak.	S8
**Figure S9.** Optimized geometries showing top as well as side views of (a) **CuDPP** and (b) **CuDPP-TCBD**.	S9
**Figure S10.** Deviation of the porphyrin 24-core atoms from the mean plane for (a) **NiDPP-TCBD** and (b) **CuDPP-TCBD**.	S11
**Table S1**. Selected bond distances and crystallographic data for **NiDPP.**	S10
**Table S2.** Selected bond lengths (Å) and bond angles (°) for the B3LYP/LANL2DZ optimised geometries of MTPPand MTPP-TCBD (M = Ni and Cu).	S11-S12

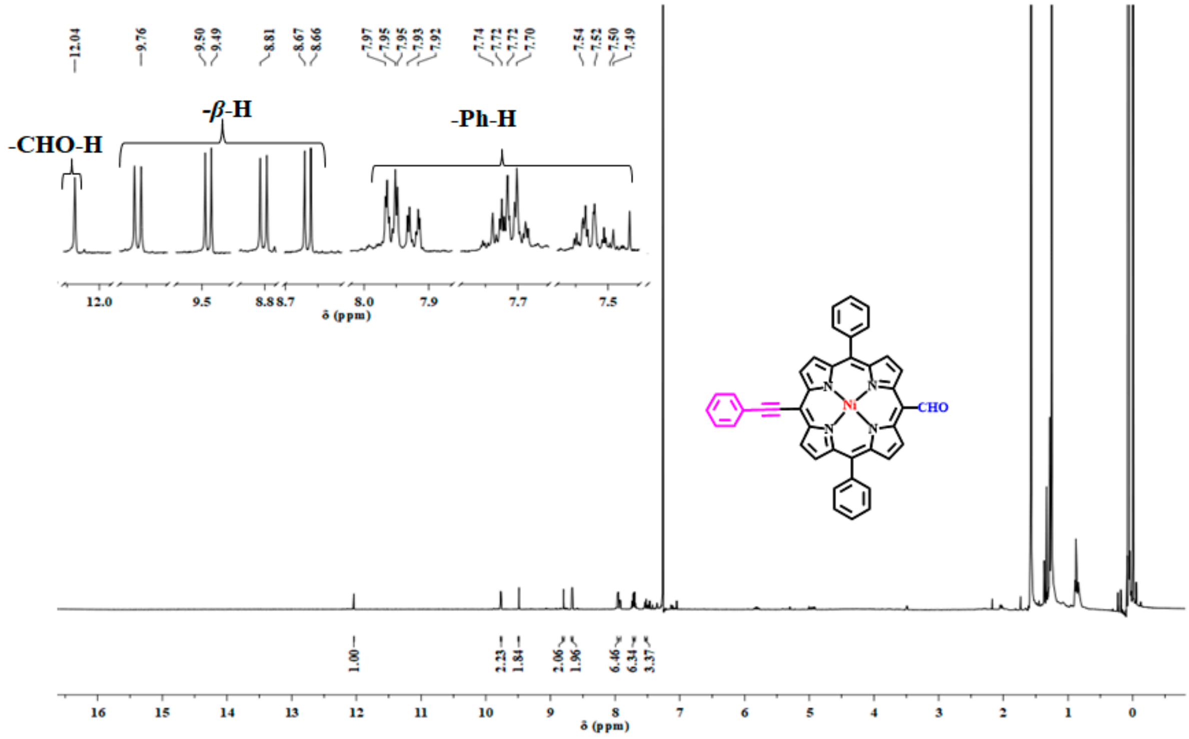

Figure S1. ^{1}H NMR spectrum of **NiDPP** in $CDCl_3$ at 298 K.

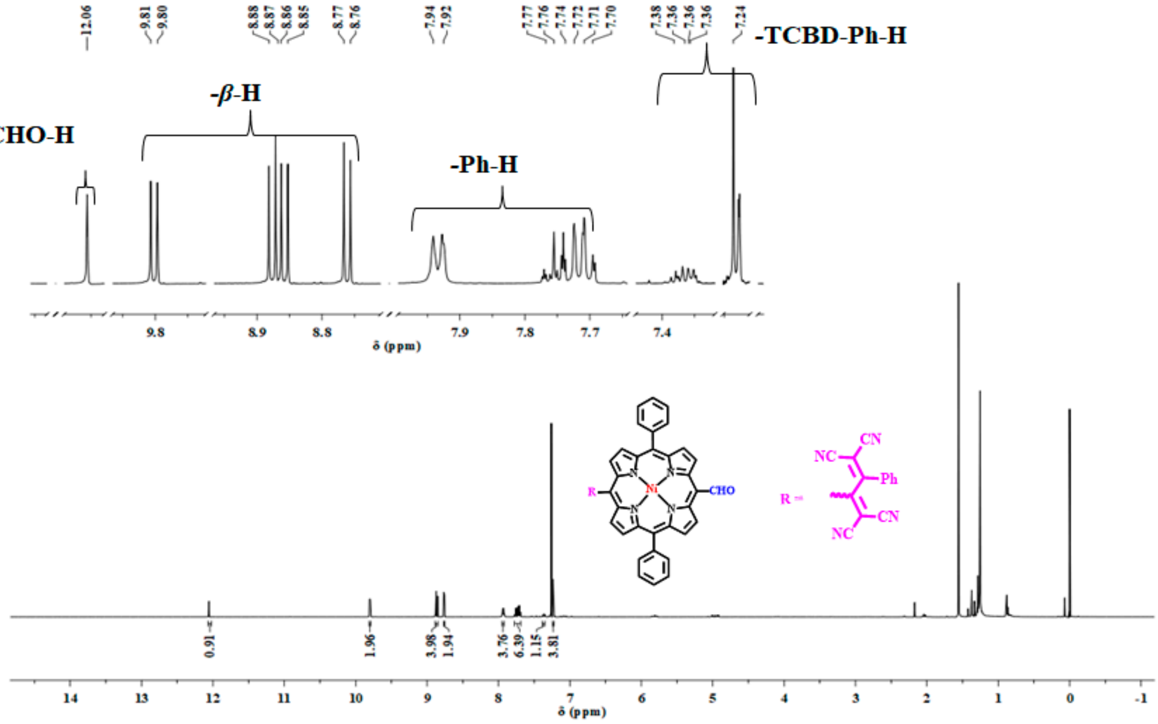

Figure S2. ^{1}H NMR spectrum of **NiDPP-TCBD** in $CDCl_3$ at 298 K.

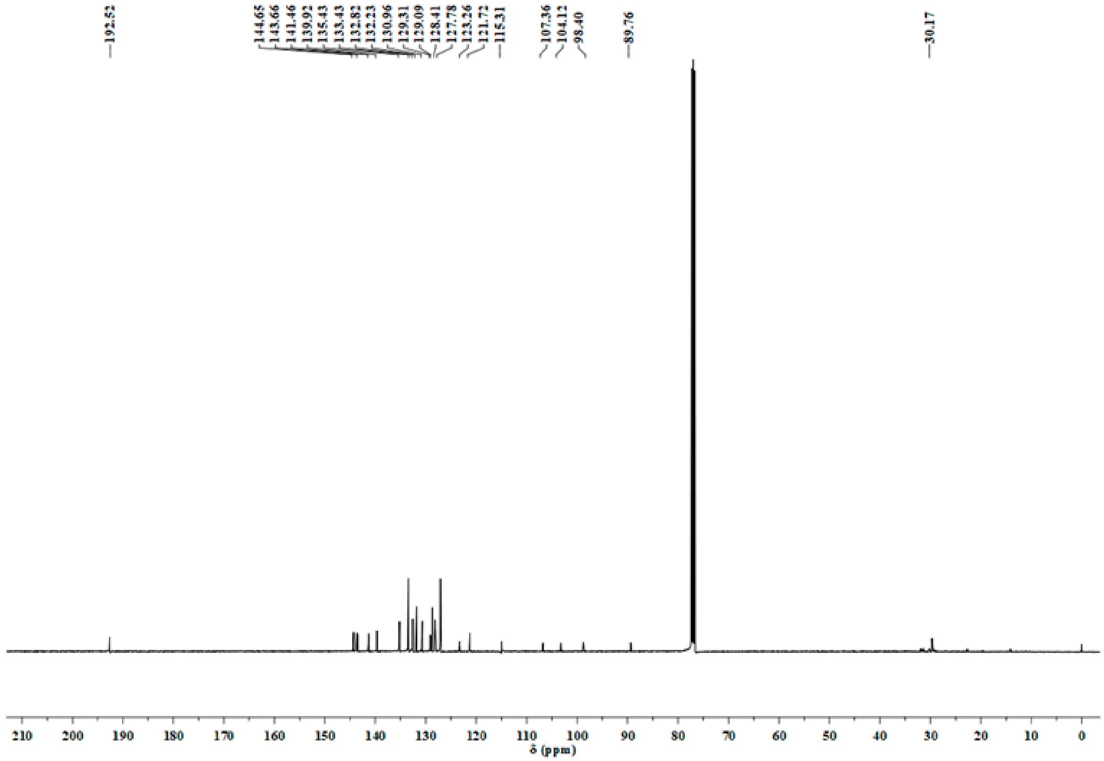

Figure S3. ^{13}C NMR spectrum of **NiDPP** in $CDCl_3$ at 298 K.

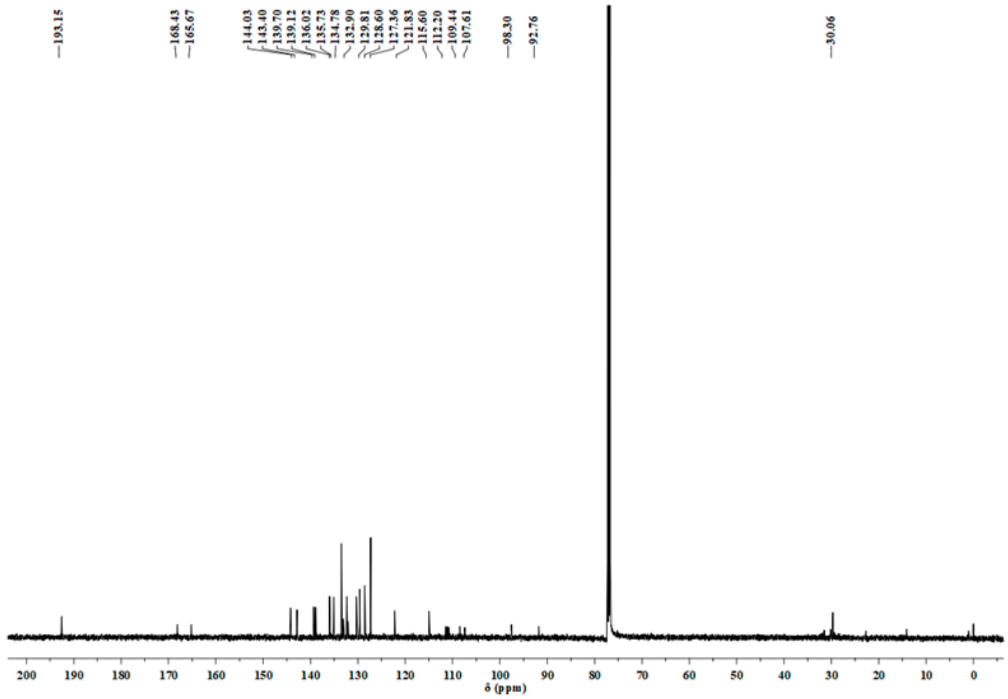

Figure S4. ^{13}C NMR spectrum of **NiDPP-TCBD** in $CDCl_3$ at 298 K.

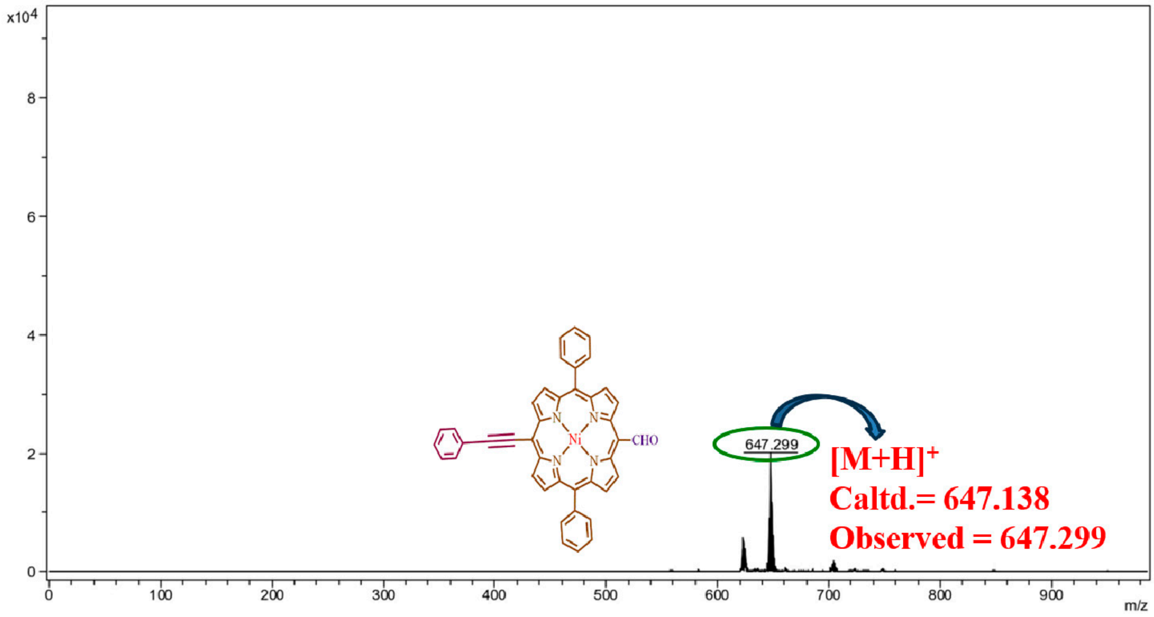

Figure S5. MALDI-TOF Mass spectrum of **NiDPP** in CH_2Cl_2 at 298 K.

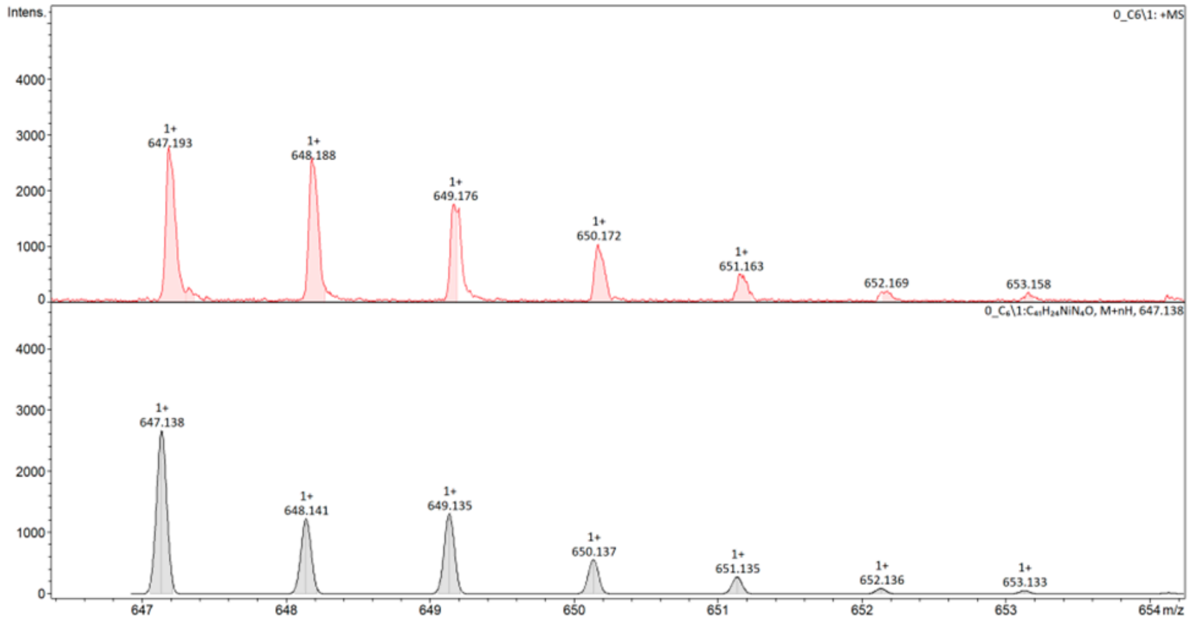

Figure S5A. Simulated (bottom) and observed (top) MALDI TOF mass spectra of expanded **NiDPP** molecular ion peak.

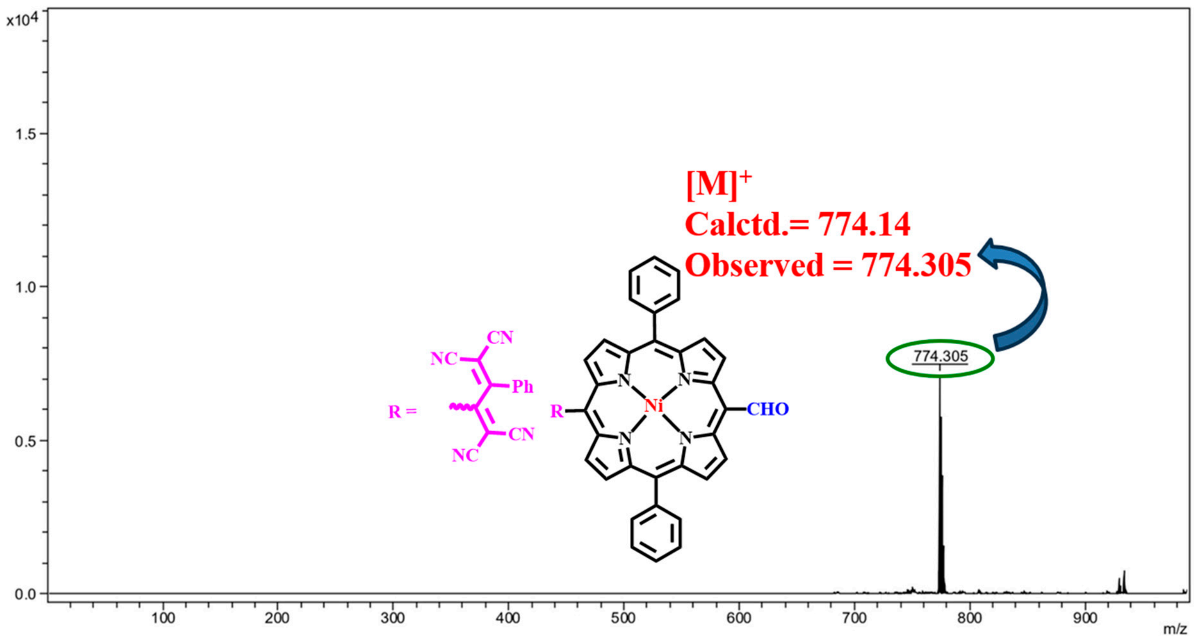

Figure S6. MALDI-TOF Mass spectrum of **NiDPP-TCBD** in CH_2Cl_2 at 298 K.

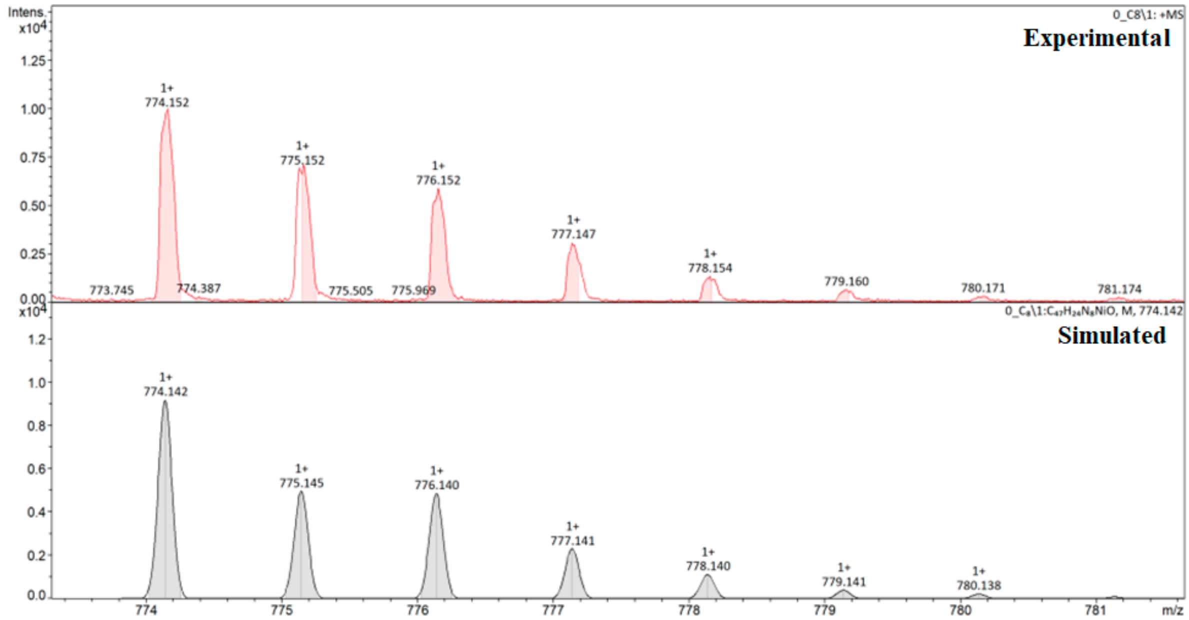

Figure S6A. Simulated (bottom) and observed (top) MALDI TOF mass spectra of expanded **NiDPP-TCBD** molecular ion peak.

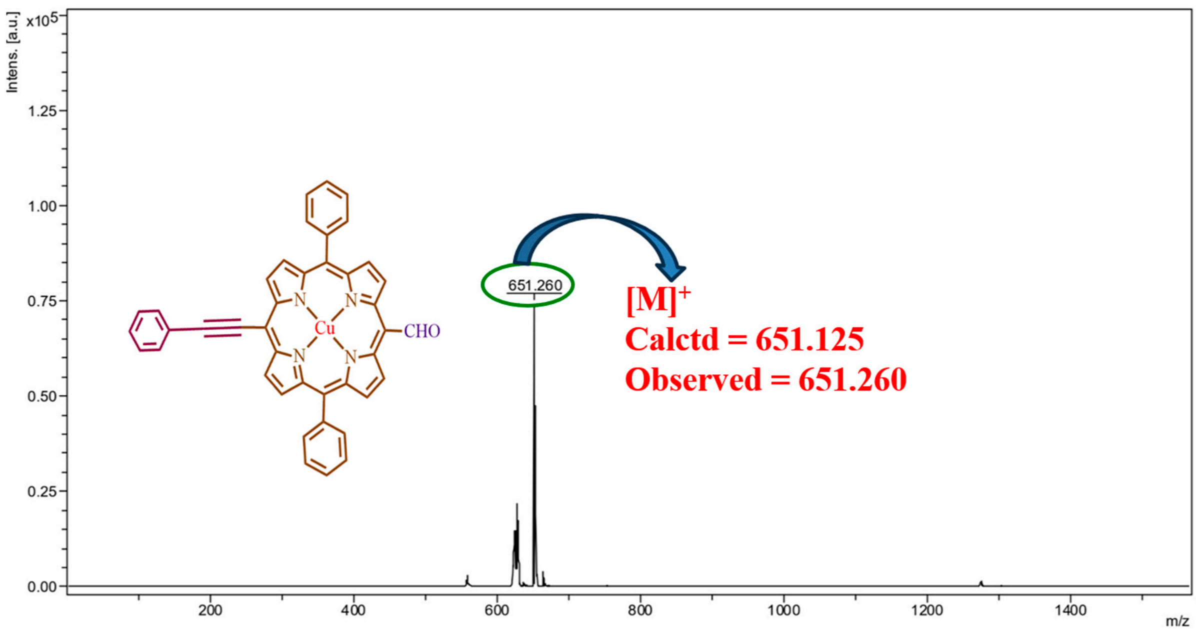

Figure S7. MALDI-TOF mass spectrum of **CuDPP** in CH_2Cl_2 at 298 K.

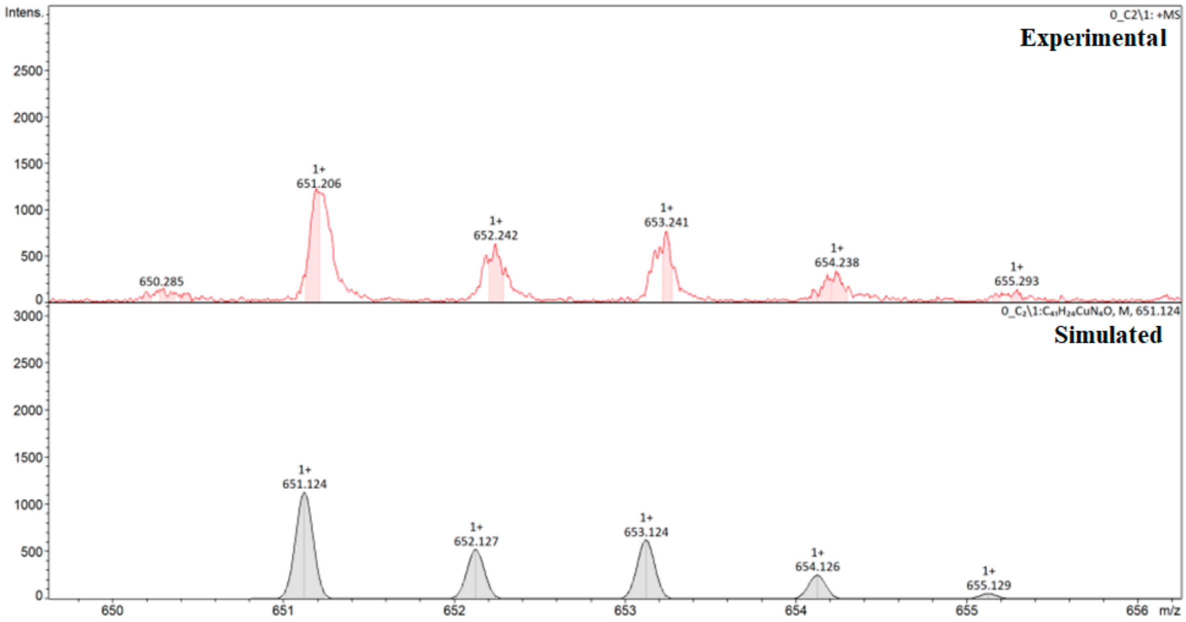

Figure S7A. Simulated (bottom) and observed (top) MALDI TOF mass spectra of expanded **CuDPP** molecular ion peak.

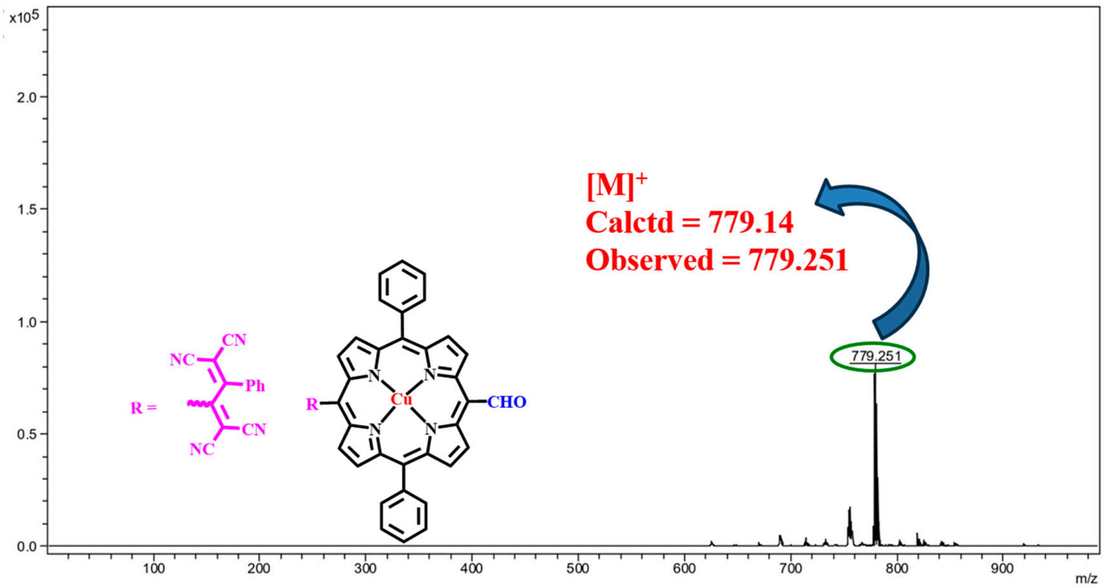

Figure S8. MALDI-TOF Mass spectrum of **CuDPP-TCBD** in CH_2Cl_2 at 298 K.

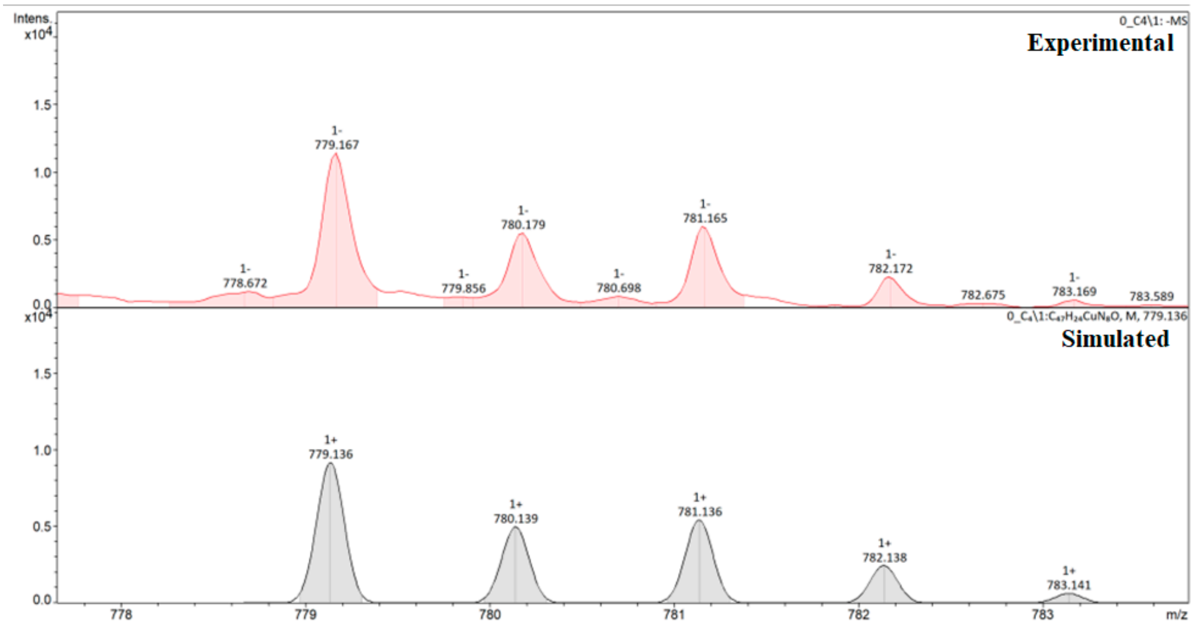

Figure S8A. Simulated (bottom) and observed (top) MALDI TOF mass spectra of expanded **CuDPP-TCBD** molecular ion peak.

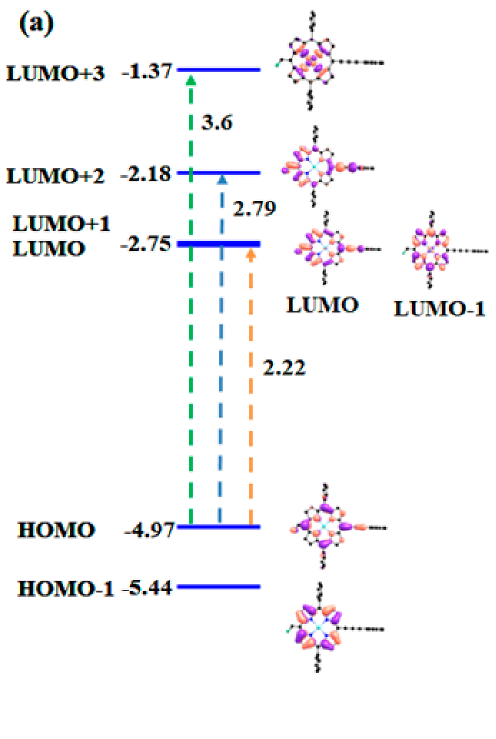

Figure S9. Optimized geometries showing top as well as side views of (a) **CuDPP** and (b) **CuDPP-TCBD.**

Table S1. Selected bond distances and crystallographic data for **NiDPP.**

	**NiDPP**
Formula	$C_{41}H_{24}N_4NiO$
Formula weight	647.35
Crystal system	triclinic
Space group	P-1
a(Å)	11.4150(11)
b(Å)	11.7115(11)
c(Å)	12.1875(12)
α (°)	108.736(3)
β(°)	106.527(4)
γ(°)	93.453(2)
Z	2
Crystal size, mm^3	0.4 × 0.2 × 0.1
Density (g/cm^3)	1.474
λ (Å)	0.71073
Temp. (K)	100
Reflection collected	64591
Independent reflections	7287
Final R indices [I = 2σ(I)]	R_1 = 0.0600, wR_2 = 0.1544
Final R indices [all data]	R_1 = 0.0739, wR_2 = 0.1650
CCDC	2518643

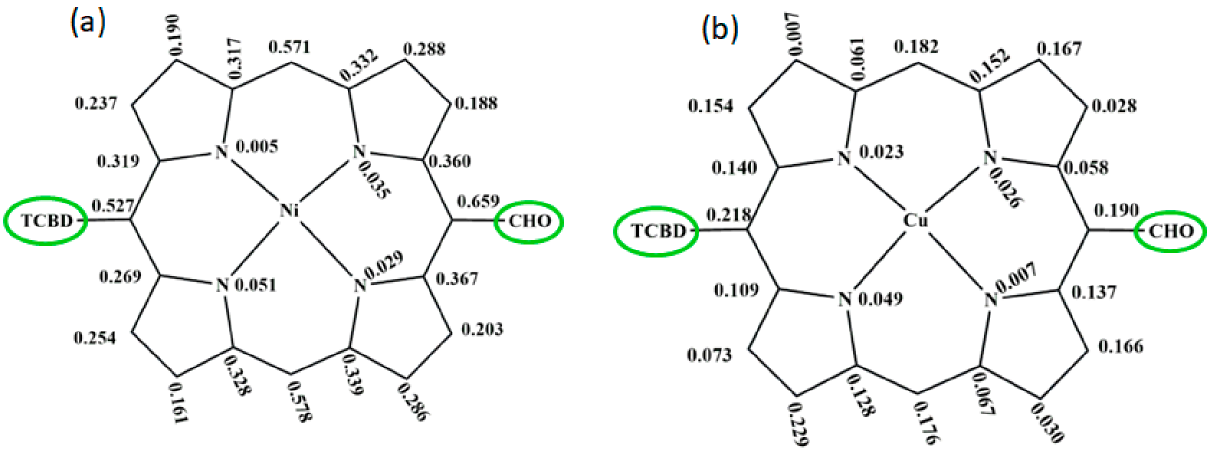

Figure S10. Deviation of the porphyrin 24-core atoms from mean plane for (a) **NiDPP-TCBD** and (b) **CuDPP-TCBD**.

Table S2. Selected bond lengths (Å) and bond angles (°) for the B3LYP/LANL2DZ optimized geometries of MDPP and MDPP-TCBD (M = Ni and Cu).

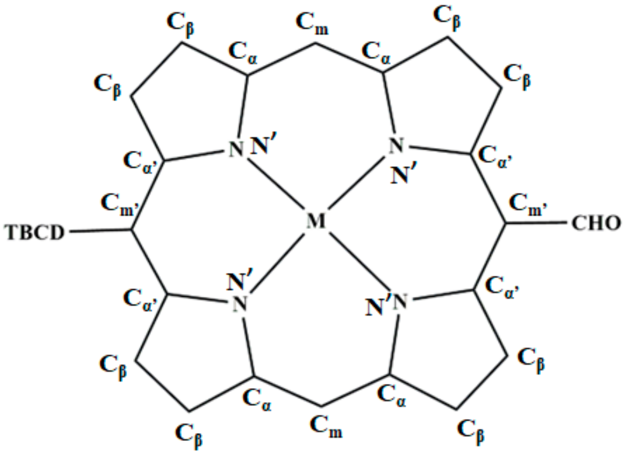

	**NiDPP**	**NiDPP-TCBD**	**CuDPP**	**CuDPP-TCBD**
	Bond Length (Å)	Bond Length (Å)	Bond Length (Å)	Bond Length (Å)
$C_{\alpha'}$-$C_{m'}$	1.410	1.409	1.427	1.416
C_{α}-C_{m}	1.402	1.403	1.405	1.406
N'-$C_{\alpha'}$	1.391	1.392	1.388	1.392
N'-C_{α}	1.388	1.399	1.403	1.401

$C_{\alpha'}$-C_β	1.452	1.454	1.452	1.459
C_α-C_β	1.451	1.451	1.450	1.453
C_β-C_β	1.372	1.371	1.373	1.369
Δ24	0.264	0.287	0.042	0.108
ΔC_β	0.219	0.226	0.033	0.107
ΔM	0.016	0.010	0.007	0.018
		Bond Angles (°)		
N'-$C_{\alpha'}$-$C_{m'}$	125.38	125.04	126.038	125.66
N'-C_α-C_m	125.77	125.89	126.445	126.63
M-N'-$C_{\alpha'}$	127.32	127.38	127.603	127.64
M-N'-C_α	127.04	126.85	126.383	126.02
N'-M-N'	89.99	90	90	90
$C_{\alpha'}$-$C_{m'}$-$C_{\alpha'}$	120.99	121.39	122.975	123.47
C_α-C_m-C_α	121.12	120.91	123.89	123.55
C_α-N'-$C_{\alpha'}$	105.60	105.71	105.99	106.24
